# *Ficus benghalensis* as Potential Inhibitor of 5*α*-Reductase for Hair Growth Promotion: *In Vitro*, *In Silico*, and *In Vivo* Evaluation

**DOI:** 10.3389/fphar.2021.774583

**Published:** 2021-12-07

**Authors:** Jawaria Iltaf, Sobia Noreen, Muhammad Fayyaz ur Rehman, Shazia Akram Ghumman, Fozia Batool, Muhammad Mehdi, Sara Hasan, Bushra Ijaz, Muhammad Safwan Akram, Haider Butt

**Affiliations:** ^1^ Institute of Chemistry, University of Sargodha, Sargodha, Pakistan; ^2^ College of Pharmacy, University of Sargodha, Sargodha, Pakistan; ^3^ Centre of Excellence in Molecular Biology, University of the Punjab, Lahore, Pakistan; ^4^ School of Health and Life Sciences, Teesside University, Middlesbrough, United Kingdom; ^5^ Department of Mechanical Engineering, Khalifa University, Abu Dhabi, United Arab Emirates

**Keywords:** androgenic alopecia, *Ficus benghalensis*, 5*α*-reductase, dihydrotestosterone, Minoxin, RP-HPLC

## Abstract

The screening of hair follicles, dermal papilla cells, and keratinocytes through *in vitro*, *in vivo*, and histology has previously been reported to combat alopecia. *Ficus benghalensis* has been used conventionally to cure skin and hair disorders, although its effect on 5*α*-reductase II is still unknown. Currently, we aim to analyze the phytotherapeutic impact of F. benghalensis leaf extracts (FBLEs) for promoting hair growth in rabbits along with *in vitro* inhibition of the steroid isozyme 5*α*-reductase II. The inhibition of 5*α*-reductase II by FBLEs was assessed by RP-HPLC, using the NADPH cofactor as the reaction initiator and Minoxin (5%) as a positive control. *In silico* studies were performed using AutoDock Vina to visualize the interaction between 5*α*-reductase II and the reported phytoconstituents present in FBLEs. Hair growth in female albino rabbits was investigated by applying an oral dose of the FBLE formulation and control drug to the skin once a day. The skin tissues were examined by histology to see hair follicles. Further, FAAS, FTIR, and antioxidants were performed to check the trace elements and secondary metabolites in the FBLEs. The results of RP-HPLC and the binding energies showed that FBLEs reduced the catalytic activity of 5*α*-reductase II and improved cell proliferation in rabbits. The statistical analysis (*p* < 0.05 or 0.01) and percentage inhibition (>70%) suggested that hydroalcoholic FBLE has more potential in increasing hair growth by elongating hair follicle’s anagen phase. FAAS, FTIR, and antioxidant experiments revealed sufficient concentrations of Zn, Cu, K, and Fe, together with the presence of polyphenols and scavenging activity in FBLE. Overall, we found that FBLEs are potent in stimulating hair follicle maturation by reducing the 5*α*-reductase II action, so they may serve as a principal choice in *de novo* drug designing to treat hair loss.

## Highlights

1) Study of the potential of *Ficus benghalensis* leaves for hair regrowth *via in vitro*, *in silico*, and *in vivo* studies.
2) The crude extract constituents inhibit steroid enzyme 5*α*-reductase II activity by binding with its active site.
3) Hair analeptics were prepared instead of direct application of crude extracts to avoid any fungal infection.
4) Photomicrographs of skin biopsies show a hair growth cycle, e.g., anagen and telogen.

## 1 Introduction

The term androgenic alopecia (AGA) refers to the patterned loss of scalp hair in men and women due to heredity and hormonal factors (Abbas et al., 2021). Hair is an indispensable structure of the body that guards the scalp, adorns human personality, and performs multiple functions such as insulation, attraction, and tangibility (Koch et al., 2020). The hair follicle (HF) cycle passes through telogen (resting phase), catagen (regression phase), and anagen (growth phase), where pigmentation and hair shaft synthesis take place (Tamura et al., 2018). Factors causing AGA are androgen hormonal imbalance, stress, genetic disorders, malnutrition, chemotherapy, 5*α*-reductase II (SRD5AII) overactivity, thyroid malfunctioning, drug addiction, ageing, and malignancy (Richards et al., 2008).

**Graphical Abstact:**
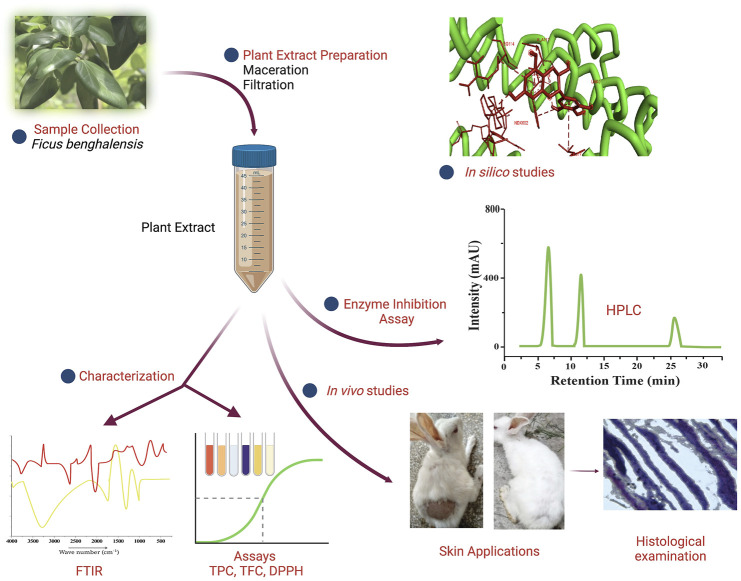


Treatment of AGA has been an open debate in clinical dermatology for many years. The risk alleles associated with AGA are located at chromosome 20p11.22 (Richards et al., 2008). “Trichoscopy” has been introduced as the first method to diagnose AGA in women (Rakowska et al., 2008). Many therapies are now available to combat baldness, like hair transplant through bioengineering (Asakawa et al., 2012), HF regeneration by rearranging stem cells (Toyoshima et al., 2012), platelet-rich plasma (PRP) (Cervantes et al., 2018), and synthetic drugs minoxidil and finasteride (Roy et al., 2008; Gregoriou et al., 2010). However, the worst dermatological effects of drugs have been observed, such as scaling, itching, and dermatitis (Choi et al., 2018). A recent study claims that ceria nanozyme-integrated microneedles can reshape the perifollicular microenvironment by reducing oxidative stress and regenerate hair at the balding site (Yuan et al., 2021). A correlation was observed between plasma micronutrients, vitamin deficiency, and hair density in non-genetic patients with AGA by direct colorimetric tests and flame atomic absorption spectrophotometry (FAAS) (Kondrakhina et al., 2020).

Human 5*α*-reductase (SDR5A) has three types of functional enzymes coded by the SRD5A gene. Among these, steroid isozyme SDR5AII (optimum pH; 5.0–5.5) plays a key role in the production of AGA by catalyzing the conversion of testosterone into more active dihydrotestosterone (DHT). The enzyme is sufficiently found in the prostate gland, epididymis, genital skin, and seminal vesicles, while the brain and liver contain fewer amounts. Moreover, DHT overexpression also causes acne, prostate cancer, and benign prostate hyperplasia (Srivilai et al., 2019). Histological studies in DHT-treated mice show delayed hair regrowth and shortened anagen (Fu et al., 2021). Computational and mutagenesis studies explained the binding interaction of SRD5AII′ to finasteride, demonstrating how the drug inhibits this integral membrane enzyme (Xiao et al., 2020).

To cure alopecia, herbs can provide nutrition, enhance scalp blood circulation, and cease DHT and SRD5A response (Kaur et al., 2020). The use of *Trigonella foenum-graecum* and *Eclipta alba* promoted hair growth in rats more efficiently than the drug (Roy et al., 2008; Imtiaz et al., 2017). Microwave-assisted solvent extraction and GC-MS reveal organic compounds in *F. benghalensis* that show an anti-inflammatory and vasodilator response (Jayasree Radhakrishnan and Venkatachalam, 2020). Under chronic stress, a high corticosterone level elongates the telogen phase due to the suppressive expression of *Gas6* (Choi et al., 2021). Interestingly, the neuromodulator effect of the methanol bark extract of *F. benghalensis* boosted memory and learning behavior and reduced stress in rats (Malik et al., 2020).

Here, we aimed to elucidate the effect of FBLEs in stimulating cell proliferation to promote hair growth (HG) in rabbits and inhibit SRD5AII activity without leaving undesired secondary effects.

## 2 Materials and Methods

### 2.1 Extract Preparation

Fresh leaves of the *F*. *benghalensis* (5 kg) were collected from Chakwal, Pakistan. The plant was authenticated by a taxonomist from the Department of Botany, University of Sargodha (UOS), Pakistan. The leaves were washed and dried in the air, followed by the production of coarse powder using a mechanical grinder. Powdered leaves (20 g) were soaked in 200 ml of either solvent—petroleum ether (PE), ethyl acetate (EtOAc), and 70% v/v aqueous ethanol (aq. EtOH) labeled as FBLE1-3. The suspensions were placed on an orbital shaker for 5 days. Later, the mixtures were filtered, subjected to evaporation, concentrated using porous aluminum foil, and finally kept at 4°C until further use (Handa et al., 2008).

### 2.2 *In Vitro* 5*α*-Reductase II Study for Hair Growth

The inhibition of the catalytic activity of SRD5AII was studied by initially mincing 4 g of the prostate gland of a male goat and crushing it under liquid nitrogen. The tissue was further homogenized using a handled homogenizer in 20 ml of sodium phosphate buffer (SPB) (pH 6.5, 50 mM) containing 1 mM EDTA (200 µl), 100 mM sucrose (64 ml), 1 mM sodium azide (200 µl), and a protease inhibitor tablet (Roche Pharma, Mannheim, Germany). Homogenate was centrifuged at 15,000 rpm for 20 min at 4°C, separating the supernatant for further use as a crude enzyme source. The soluble protein was tested by Bradford assay (Rehman et al., 2009).

For the reverse-phase high-performance liquid chromatography (RP-HPLC; Shimadzu, Japan), Testovirone ampoule (testosterone enanthate, 250 mg/ml; Bayer Pharma, Leverkusen, Germany) was taken as standard. Six reaction mixtures (RMs) were prepared as positive control (PC), reaction control (standard), complete reaction (internal standard, IS), and others with FBLEs (1–3) (Table 1). The RMs were incubated at 37°C for 30 min, and the reaction was terminated by adding 2 ml of EtOAc followed by the addition of 150 µl prednisolone 250 μg/ml ethanol as an IS. The RMs were mixed for 1 min and centrifuged at 5,000 rpm (10 min, 4°C). Subsequently, the water phase was frozen at −80°C, and the organic layer was decanted and evaporated. The concentrated residue (1.5 ml) was later diluted in 3 ml methanol. Finally, RMs were syringe filtered (0.2 µm) before HPLC run to avoid contamination (Sher et al., 2015; Kumar et al., 2011).

**TABLE 1:** Composition of RMs for *in vitro* enzyme activity assay.

Sr.no	Reagents	Standard (µL)	IS (µL)	PC (µL)	1–3 (µL)
1	FBLEs	−	−	−	200
2	SPB	500 µL	500 µL	500 µL	500 µL
3	10% alc. testoviron	−	150 µL	150	150
4	Crude enzyme	500 µL	500 µL	500 µL	500 µL
5	NADPH	−	200	200	200
6	Testosterone enanthate	10	−	−	−
7	Minoxin^®^ (5%)	−	−	200	−

The RP-HPLC was performed using Chromatographic Column C18-ODS type (250 mm × 4.6 mm), maintaining system conditions at 40°C and flow rate 10 μl/min for 30 min, while absorbance was recorded at 254 nm. Isocratic elution was performed, making a mobile phase with methanol and water (80:20) and filtering it to remove any contamination. The percentage inhibition of SRD5AII was observed from the peak height and the peak area ratio (r) applying the following formula (Kumar et al., 2011): $\left(\text{\%}\right)=\frac{r\;sample\;-\;r\;reaction}{r\;standard\;-\;r\;reaction}\times 100\;$ r = $\frac{\text{Area of sample}}{\text{Area with least value}}$ ; Peak area (mm^2^) = height × width_1/2_.

### 2.3 Molecular Docking

To determine the ligand–receptor binding interactions, we docked SD5ARII with minoxidil and reported bioactive constituents: (-)-Gallocatechin, Rhein, and Mucusoside of FBLEs using AutoDock Vina and AutoDock Tools 1.5.6. The X-ray crystal structure of the target mammalian’s SD5ARII with a resolution of 2.8 Å was retrieved from Protein Data Bank (PDB ID: 7BW1). After docking, the binding energies (BEs) were noted, and the lowest values were chosen to view the interactions in PyMol (Bhaskara Rao et al., 2014; Hassan et al., 2020; Bilal et al., 2021; Rehman et al., 2021).

### 2.4 *In Vivo* Study for HG

The female albino rabbits (6–11 months old; 1–2 kg) were purchased from the University of Veterinary and Animal Sciences, Lahore, Pakistan. Prior to proceeding further, rabbits were authorized by the ethical committee of the College of Pharmacy, UOS, providing approval no. 70B18 IAEC/UOS. Animals were acclimatized for 3 days providing standard food and water in an animal housing facility at the College of Pharmacy, UOS. Rabbits were divided into eight groups, shaved at the dorsal side area (5 × 4 cm), using a razor, and left for 24 h to observe any edema or erythema. HG formulations (500 µl) were sprayed once a day on the nude area, and eventually HG was observed. On the 28th day, rabbits were sacrificed, and skin biopsies were performed while skin tissues were kept in 10% formalin for histological study (Roy et al., 2008; Imtiaz et al., 2017). Ten hair strands were randomly plucked every week, and an average length of each hair was noted in mm using a digital Vernier caliper (Adhirajan et al., 2003). Histology was performed using subcutaneous hematoxylin and eosin staining (Lin et al., 2015) to analyze HFs, observed at ×10 magnification using a digital microscope (Bresser, Rhede, Germany).

#### 2.4.1 Hair Growth Formulations

The HG formulations were prepared by mixing dilute FBLEs (10 ml), EtOH (7.5 ml), and 32 ml of distilled water, followed by ultrasonication. Later, it was syringe filtered (0.24 µm) to avoid any microbial infection on the animal skin and noted pH. Minoxin^®^ 5% (Brookes Pharma, Karachi, Pakistan) was used as PC, whereas PE, EtOAc, and 70% aq. EtOH were used as negative controls (NCs) (Imtiaz et al., 2017).

### 2.5 Qualitative Phytochemical Analysis

Phytochemical analysis was performed to analyze various phytoconstituents in crude extracts like phenolic compounds and tannins, alkaloids, saponins and glycosides, terpenoids and steroids, anthocyanins, flavonoids, coumarins, and quinones (Harborne, 1998). The results of qualitative analysis were noted with color change, foamy appearance, or precipitates formation.

### 2.6 Characterization of FBLEs

The FBLEs were analyzed for calcium, iron, zinc, and potassium using FAAS. The powdered leaves (50 mg) were digested using 15 ml of HCl and HNO3 (3:1) to extract metal ions. The acid mixture was poured into the beaker having powdered leaves and placed on a hot plate until the sample got mixed thoroughly. The acidic mixture was allowed to cool, then filtered, and finally diluted up to 100.0 ml using distilled water. Stock solutions of 3, 6, 9, 12, and 15 ppm of metal salts were prepared for mineral detection, and concentrations were noted in mg/g (Ahmad et al., 2014). Chemical characterization of FBLEs was done through Fourier transform infrared (FTIR) spectroscopy. The greasy extracts were dried using KBr pellets and analyzed in the mid-range, 4,000–600 cm-1 (Gopukumar et al., 2016).

### 2.6 Antioxidant Activity

Total polyphenol content (TPC) was estimated by modifying a method (Abbas et al., 2021), taking gallic acid as a standard, and FBLEs (50 mg) diluted in 5 ml methanol were used to determine TPC. The absorbance was noted at 765 nm in triplicate. Total flavonoid content (TFC) was estimated by aluminum chloride colorimetric assay, taking catechin as a standard, using FBLE dilutions prepared for TPC. Each reading was measured in triplicate at 510 nm.

### 2.7 DPPH Assay

Radical scavenging activity (RSA) was assessed using DPPH (1,1-diphenyl-2-picrylhydrazyl) assay, taking 100 µl FBLE dilutions, added to 3 ml of DPPH (0.1 mM), followed by incubation at room temperature in the dark for 30 min. The absorbance was recorded in triplicate at 517 nm while taking 3 ml of DPPH as control (Abbas et al., 2021). Data were recorded as % RSA, and IC_50_ (minimum inhibitory concentration) was calculated using MS Excel 2016. The percentage of RSA was calculated using the following formula;

$$\text{RSA}\left(\text{\%}\right)=\frac{Abs\;control\;–\;Abs\;sample}{Abs\;control}\times 100$$

### 2.8 Statistical Analysis

The statistical analysis was scrutinized by one-way ANOVA *via* Tukey test using SigmaPlot ver. 14.0. The Pearson correlation coefficient (p) was applied to correlate HG results among all test groups at significance level *p* < 0.05 or highly significant *p* < 0.01 using SPSS version 21.

## 3 Results

### 3.1 Percentage Yield

The percentage yield of FBLEs (PE, EtOAc, and 70% aq. EtOH) was calculated as 3.9%, 4.8%, and 20.6%, respectively.

### 3.2 *In Vitro* Enzyme Study

The quantified protein from the male goat prostate gland was 7.99 mg/ml of BSA. In the RP-HPLC charts, a prominent peak (b) at retention time (RT) around 6 min was entitled as the testosterone’s peak (Figure 5D). Results are justified by the peak height intensity (mAU), which determines how much testosterone has been left behind in the RM. The results of (r) and peak height revealed more unconverted testosterone in hydroalcoholic FBLE, favoring sufficient percentage inhibition for SRD5AII, than Minoxin 5% (Table 2). No nicotinamide adenine dinucleotide hydrogen phosphate (NADPH) was added in the “reaction control” to compare the peak height of the PE 1-3 and PC. However, the minimum peak height a) observed in the complete reaction with IS indicated more testosterone conversion to DHT. A delayed peak c) refers to SRD5AII due to the hydrophobic nature of both steroid enzyme and stationary phase.

**TABLE 2:** Peak area ratio (r) and percentage inhibition of SRD5AII by RMs.

Peak	Area (mm^2^)	Ratio	% inhibition
Standard	330	2.5	−
IS	132.6	1.0	−
Minoxin 5%	233.3	1.76	50.6
PE 1	225.5	1.7	46.6
PE 2	215.1	1.62	41.3
PE 3	274.5	2.07	71.3

### 3.3 Molecular Docking

**FIGURE 1:**
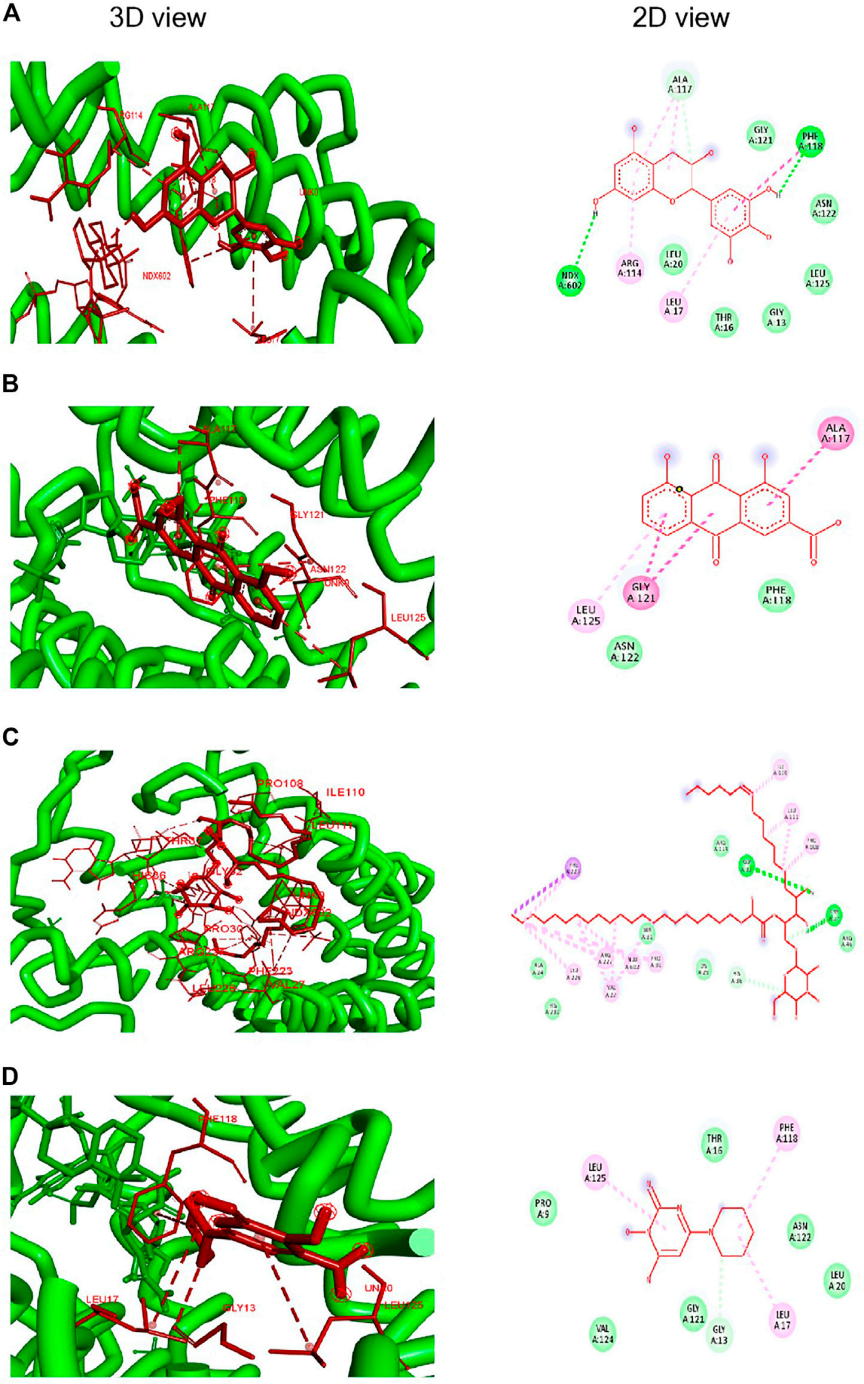
Molecular docking of SD5ARII with the ligands. **(A)**–(–Gallocatechin); **(B)** Rhein; **(C)** Mucusasoide, and **(D)** Minoxidil.

Docking studies showed a strong binding interaction of Rhein (BE; −7.4 kcal/mol) with the enzyme’s active sites, revealing a prompt inhibitory potential against SD5ARII. In comparison, the binding energies (Bes) of phytoconstituents of crude FBLEs showed significant interactions with the receptor protein than Minoxidil. Figure 1 and Table 3 show protein–ligand interactions and values of their BEs in Kcal/mol and type of binding interaction.

**TABLE 3:** Docking of bioactive compounds reported in *Ficus benghalensis* leaf extracts.

Ligands	Docking score (kcal/mol)	Residues	Interaction
(-)- Gallo catechin	−6.9	NDX A:602	Conventional H-bond
		PHE A:114	Conventional H-bond and Pi–Pi T shaped
		ARG A:114	Pi-alkyl
		LEU A:17	Pi-alkyl
		ALA A:117	Pi-alkyl and C–H bond
Rhein (1,8-OH,3-COOH)	−7.4	ALA A:117	Amide-Pi-stacked
		GLY A:121	Amide-Pi-stacked
		LEU A:125	Pi-alkyl
Mucusoside	−4.5	GLY A:32	Conventional H-bond
		THR A:37	Conventional H-bond
		PHE A:223	Pi-sigma and alkyl
		HIS A:36	C–H bond
		ILE A:110	Alkyl
		LEU A:110	Alkyl
		PRO A:108	Alkyl
		PRO A:30	Alkyl
		NDX A:602	Alkyl
		VAL A:27	Alkyl
		ARG A:227	Alkyl
		LUE A:26	Alkyl
Minoxidil	−6.0	GLY A:13	C–H bond
		LEU A:125	Pi-alkyl
		LEU A:17	Pi-alkyl
		PHE A:118	Pi-alkyl

### 3.4 *In Vivo* Analysis

**FIGURE 2:**
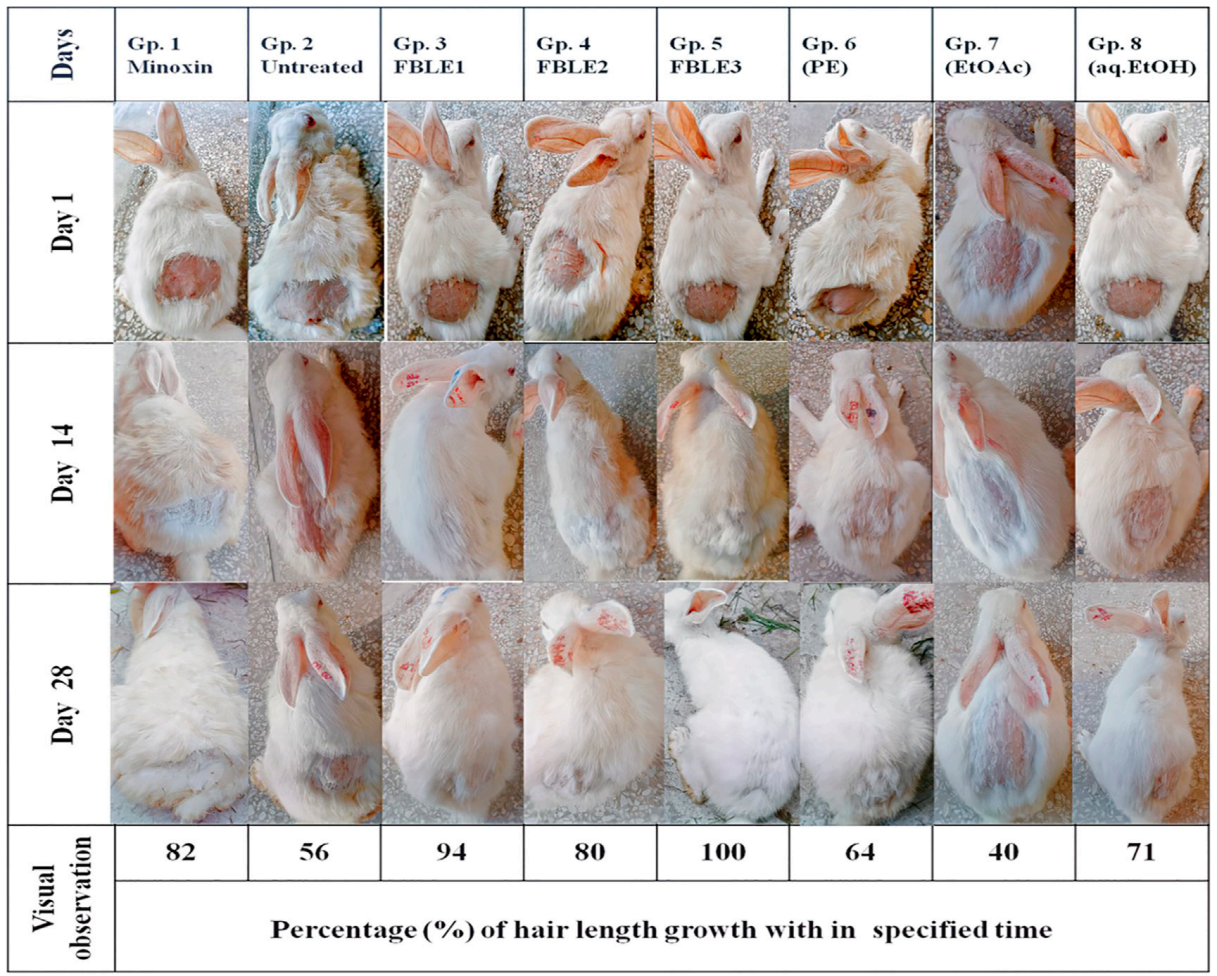
*In vivo* hair growth progress after 28 days of treatment.

**FIGURE 3:**
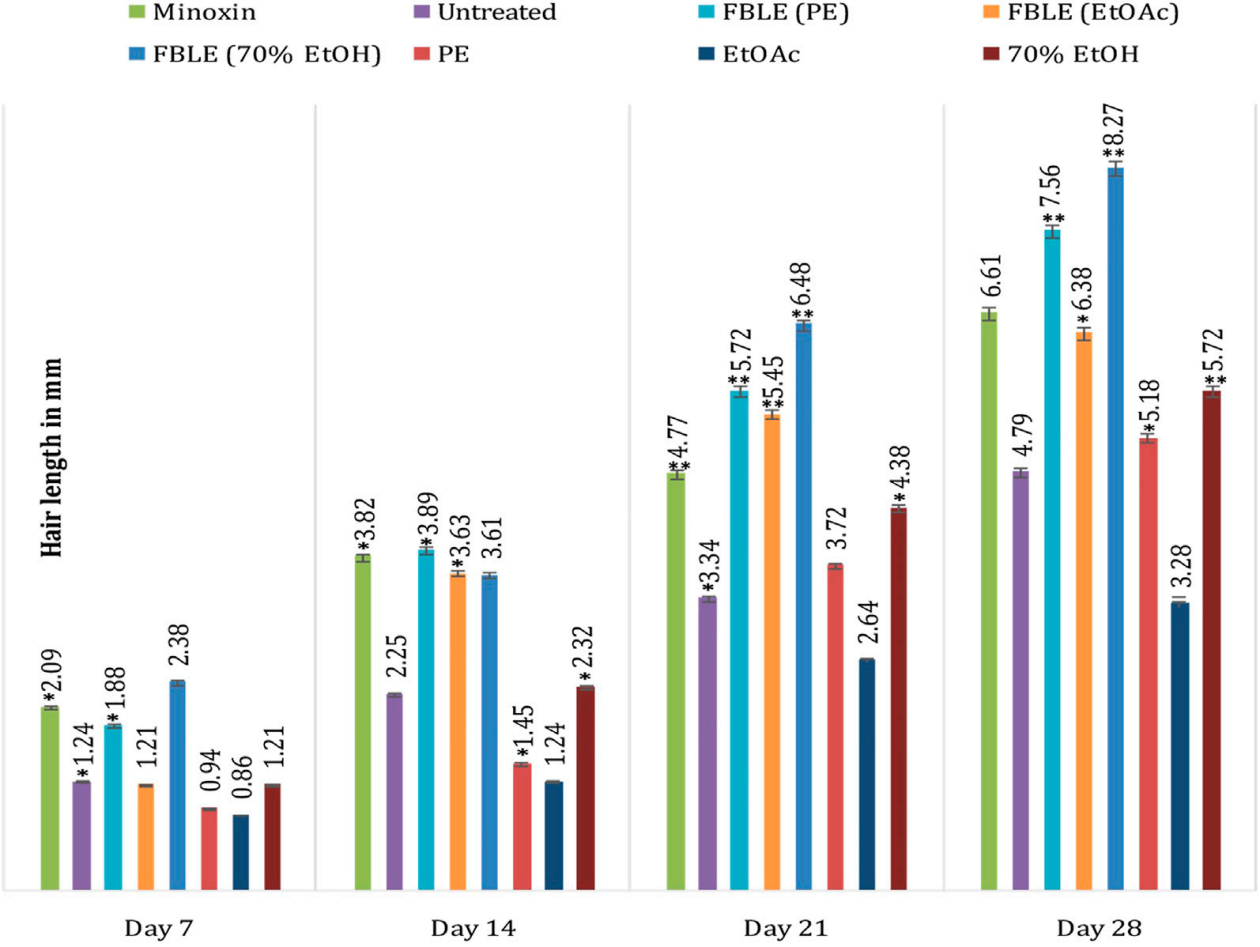
Improvement in hair growth within due time shows the weekly increase in hair length. Mean ± SD * Significant at *p* < 0.05; **p < 0.01.

**FIGURE 4:**
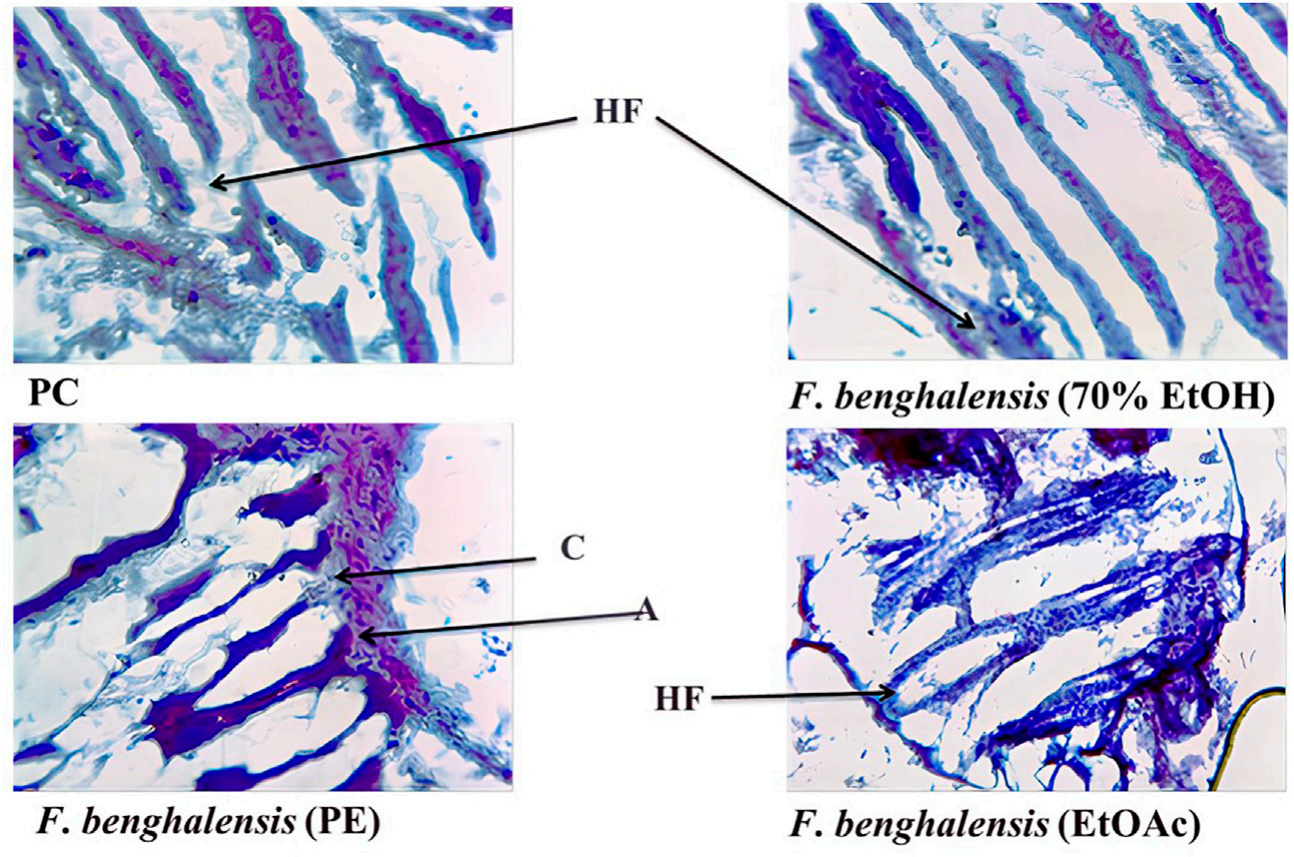
Photomicrographs of the longitudinal section of skin biopsies under digital microscope × 10 resolution; PC (Minoxin 5%) and crude FBLE extracts depict HF= Hair Follicle, anagen **(A)**, and C= catagen **(B)**.

No skin irritation was observed in the rabbits, and rapid hair regrowth was noticed in the first week that varied subsequently Figure 2. The hair texture appeared to be rough and weak in PC and NCs but smooth in others. The average hair length confirms that weekly HG was approximately 1–2 mm Figure 3. The Pearson correlation (*p* < 0.05) revealed an exceptional HG rate in group 5. From the photomicrographs of the longitudinal section, HF phases and DPCs were observed in treated tissues (Figure 4). The pH outcome was 7.6, 4.3, and 6.4 for FBLE1-3, respectively.

### 3.5 Qualitative Phytochemical Analysis

The screening of the preliminary phytochemicals has shown the presence of carbohydrates, proteins, phenolic compounds, flavonoids, alkaloids, saponins, glycosides, steroids, and tannins. The results of all phytochemicals are represented in Table 4.

**TABLE 4:** Preliminary phytochemical screening of extracts of *Ficus benghalensis*.

Phytochemicals	Phytochemical tests	Observation	(Pet. ether)	(EtOAc)	(EtOH)
Proteins	1) Ninhydrin test	Blue/violet color/ppt	+++	++	−
	2) Biuret test		+++	++	+
Carbohydrates	1) Fehling’s test	Brick red ppt	+	++	+++
	2) Benedict’s test		+	++	+++
Alkaloids	Wagner’s test	Reddish-brown	+	+	++
Saponins	Foam test	Foam on surface	+++	+	−
Phenols and tannins	Ferric chloride test	Bluish-green	+	+	+
Terpenoids and steroids	Salkowski test	Reddish-brown	++	+	+++
Glycosides	A) Keller–Kilani test	Brown ring/reddish-brown	++	+	+++
	B) Salkowski test		++	+	+++
Anthocyanins	Hydrochloric acid test	Blue color	++	+	+
Quinones	Sulfuric acid test	Red color	+	+	+
Flavonoids and coumarins	Sodium hydroxide test	Yellow color	+	+	++

+Slightly present, ++ present, +++ strongly present, − absent.

### 3.6 FAAS and FTIR Analysis

**FIGURE 5:**
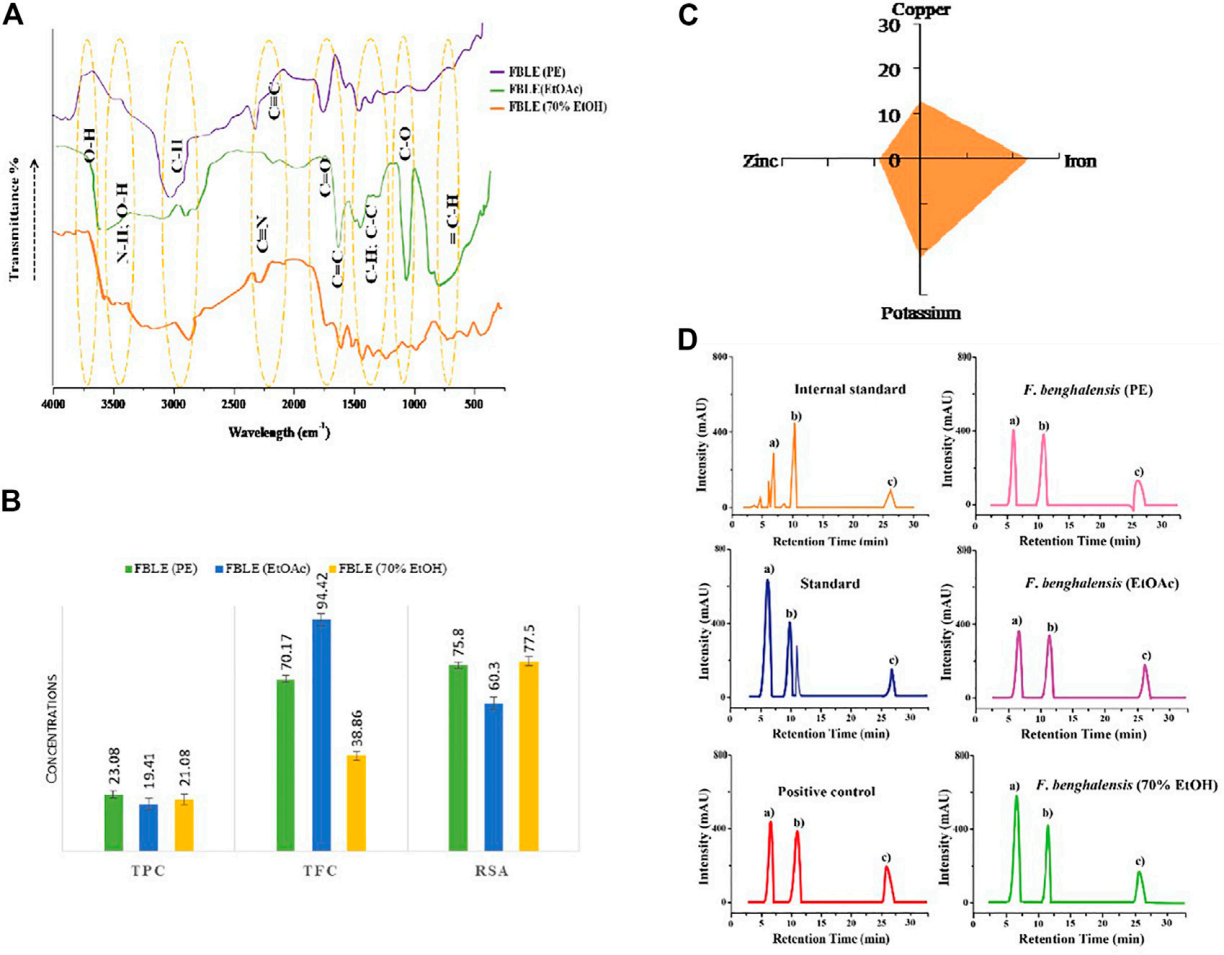
Characterization of FBLEs and RP-HPLC analysis. In **(A),** FTIR fingerprints show the functional groups; **(B)** presence of essential minerals in dried leaves; **(C)** depicts antioxidants present in FBLEs. Values are mean ± SD. **(D)** demonstrates how much FBLEs are active in limiting SRD5AII activity. The peak **(B)** in RP-HPLC graphs indicates the availability of testosterone in RMs; the same peak is represented by **(A)** in the graph labeled as “IS”.

FAAS showed the mineral concentrations in mg/g (copper = 13; zinc = 9; iron = 23.4; potassium = 21.8) found in powdered leaves. In FTIR fingerprints, peaks are attributed to stretching and bending vibrations, characterizing functional groups in essential metabolites (Figures 5A,C).

### 3.7 Antioxidant Activity

FBLEs showed concentrations for TPC, TFC, and RSA, presented in Figure 5B.

### 3.8 DPPH Scavenging Activity

Different concentrations of FBLEs against DPPH were tested to find the inhibitory potential of antioxidants present in the crude extracts. It has been observed that %RSA increased as the concentrations of FBLEs increased. Data were recorded as mean ± SD. Table 5 shows the maximum %RSA and IC_50_ values for FBLEs.

**TABLE 5:** DPPH radical scavenging and antioxidant capacity of FBLEs.

Plants extracts	TPC (mg GAE/g)	TFC (mg CE/g)	% RSA	IC_50_
FBLE (PE)	23.1 ± 0.33^***^	70.2 ± 0.72^***^	75.8 ± 0.01	2.57 ± 0.05
FBLE (EtOAc)	19.4 ± 0.60^***^	94.4 ± 0.51^***^	60.3 ± 0.01	14.1 ± 0.01
FBLE (aq. EtOH)	21.1 ± 0.79^***^	38.9 ± 0.36^***^	77.5 ± 0.01	18.9 ± 0.01

Values are mean ± SD; *n* = 3; ^***^significant at *p* < 0.001 applying the Tukey test.

## 4 Discussion

Androgenic alopecia (AGA), a leading disorder in both genders, plays a role in building psychological trauma (Koch et al., 2020), demanding a detailed study to cope with the disease. In the current study, *in vitro* and *in vivo* effects of FBLEs were tested for promoting HG in rabbits. Previously, females suffering from AGA were tested for aromatase mRNA levels by RT-PCR from plucked hair strands from the top of the scalp. Most women had low levels of aromatase mRNA and high levels for SRD5AI, II, and III (Sánchez et al., 2018). The *in vitro* evaluation of valproic acid in cultured human DPC decreased the level of *β*-catenin and increased anagen induction in mice (Jo et al., 2013).

The prostate gland holds sufficient amount of isozyme SRD5AII, and its homogenate explains the phenomena of testosterone’s conversion into DHT in RM (Steers, 2001). To study the inhibitory activity of SRD5AII, 17 hydroalcoholic Thai plant extracts were examined using HPLC analysis (Kumar et al., 2011). The *in vitro* study describes limiting the conversion of testosterone to DHT by lowering the catalytic activity of SRD5AII. In RP-HPLC, the peak intensity indicates more testosterone level in the RMs and thus more inhibition of SRD5AII by *Ocimum basilicum* (Kumar et al., 2011); in comparison, FBLEs were found to inhibit SRD5AII (Figure 5D; Table 2) significantly. We assumed that the peak at (RT = 11.8 min) is castor oil present in the standard and testosterone. *In silico* molecular docking of phytochemicals of FBLEs was performed to observe the inhibition of acetylcholinesterase against Alzheimer’s (Hassan et al., 2020).

The proliferation of dermal papillae cells (DPCs) and keratinocytes contributes a pivotal role in extending HF anagen while protecting the skin tissues (Madaan et al., 2017; Bejaoui et al., 2021). DPCs, the specialized markers, are cultured as a therapeutic tool during hair constitution assays to expand HFs and sustain hair inductivity (Yang and Cotsarelis, 2010). The *in vivo* studies claim that HG promoted the potential of shikimic acid and cilostazol on C57BL/6 mouse and *ex vivo* DPC proliferation of human HF by upregulating vasodilation and HG factors through kinase assays (Choi et al., 2018; Choi et al., 2019). *Eclipta alba* has imparted more HG in rats than minoxidil (Roy et al., 2008). The FBLEs promoted cell proliferation and prolonged anagen, indicated by an increase in the HG rate (Figures 3,4). Researchers have reported that a minor acidic pH is essential for healthy HG (Dias et al., 2014); pH results in the range between 4 and 8 for FBLE1-3 favor the study.

A lack of minerals could risk for producing alopecia in women (Almohanna et al., 2019); AGA male patients had insufficient zinc and copper in hair, serum, and urine (Ozturk et al., 2014). Concentrations in powdered leaves (0.01–0.6 mg/100 g) were found to be sufficient (as daily recommended dose) (Figure 5C) as compared to other species of *Ficus* (Wangkheirakpam and Laitonjam, 2012). The DPPH assay was chosen as a reliable method to find the potential of antioxidants to convert DPPH solution into non-radical DPPH. The hydro-alcoholic bark extract of *F. benghalensis* showed TPC (23.2 ± 0.6), TFC (100.24 ± 4.21), and RSA (45.73 ± 1.17) µg/ml (Wangkheirakpam and Laitonjam, 2012), respectively. A significant %RSA (85.20 ± 0.96%) was shown by the fruit extract of *F. auriculata* (Shahinuzzaman et al., 2021). Our findings revealed competitive outcomes for antioxidants in FBLEs mentioned in Table 5 and Figure 5B. Leaves of *F. benghalensis* yield *β*-amyrin along with psoralen, *β*-sitosterol, bergapten, friedelin, glycyl-d-asparagine lupeol, quercetin-3-galactoside, rutin, and taraxosterol all come under the classes of phytoconstituents (Murti et al., 2010). Further, a qualitative assessment of secondary metabolites and reported compounds of FBLEs we used for *in silico* analysis favors the confirmation of the presence of essential components in the FBLEs.

Altogether, FBLEs prepared using maceration upregulated HG in rabbits without leaving skin rash and reducing the biocatalytic potential of SRD5AII, thus depriving the conversion of testosterone to more potent DHT. The study has shown the possible binding interaction of phytochemicals with SRD5AII, inhibiting the enzyme’s activity. Histological findings depicted clear anagen in the particularly hydroalcoholic FBLE-treated group, validating visually enhanced HG in the animal model. Hence, the conclusion favors that phytoconstituents of *F. benghalensis* could serve as future drug candidates.

## Data Availability

The raw data supporting the conclusion of this article will be made available by the author, without undue reservation.
